# Human papillomavirus testing on self‐collected samples to detect high‐grade cervical lesions in rural Bhutan: The REACH‐Bhutan study

**DOI:** 10.1002/cam4.5851

**Published:** 2023-03-31

**Authors:** Gary M. Clifford, Iacopo Baussano, Daniëlle A. M. Heideman, Sangay Tshering, Tashi Choden, Fulvio Lazzarato, Vanessa Tenet, Silvia Franceschi, Teresa M. Darragh, Tashi Tobgay, Ugyen Tshomo

**Affiliations:** ^1^ Early Detection, Prevention and Infections Branch International Agency for Research on Cancer (IARC/WHO) Lyon France; ^2^ Department of Pathology Amsterdam UMC location Vrije Universiteit Amsterdam Amsterdam The Netherlands; ^3^ Cancer Center Amsterdam, Imaging and Biomarkers Amsterdam The Netherlands; ^4^ Department of Obstetrics & Gynaecology Jigme Dorji Wangchuck National Referral Hospital Thimphu Bhutan; ^5^ Department of Pathology & Laboratory Medicine Jigme Dorji Wangchuck National Referral Hospital Thimphu Bhutan; ^6^ Cancer Epidemiology Unit “Città della Salute e della Scienza” Hospital Turin Italy; ^7^ Centro di Riferimento Oncologico (CRO), IRCCS Aviano Italy; ^8^ University of California San Francisco California USA

**Keywords:** cancer prevention, screening, viral infection, women's cancer

## Abstract

**Background:**

“REACH‐Bhutan” aimed to evaluate the feasibility and clinical performance of a community‐based screening program for cervical cancer in rural Bhutan using self‐collected samples for high‐risk human papillomavirus (HR‐HPV) testing.

**Methods:**

In April/May 2016, 2590 women aged 30–60 years were screened across rural Bhutan by providing a self‐collected sample for *care*HPV testing. All *care*HPV‐positive women, plus a random sample of *care*HPV‐negative women, were recalled for colposcopy and biopsy. Self‐samples also underwent GP5+/6+ polymerase chain reaction (PCR)‐based HR‐HPV DNA detection and genotyping. Cross‐sectional screening indices were estimated against histological high‐grade squamous intraepithelial lesions or worse (hHSIL+), including imputation of hHSIL+ in women without colposcopy.

**Results:**

HR‐HPV positivity was 10.2% by *care*HPV and 14.8% by GP5+/6+ PCR. Twenty‐two cases of hHSIL+ were histologically diagnosed, including one invasive cancer; an additional 7 hHSIL+ were imputed in women without colposcopy. HR‐HPV testing by GP5+/6+ showed higher sensitivity for hHSIL+ (89.7%, 95% CI 72.6–97.8) than *care*HPV (75.9%, 95% CI 56.5–89.7). Negative predictive value was also slightly higher for GP5+/6+ (99.9%, 95% CI 99.6–100) than *care*HPV (99.7%, 95% CI 99.4–99.9). Specificity, however, was lower for GP5+/6+ (86.1%, 95% CI 84.6–87.4) than *care*HPV (90.6%, 95% CI 89.4–91.7), as was positive predictive value (6.9%, 95% CI 4.5–9.9 vs. 8.5%, 95% CI 5.4–12.6). Of 377 HR‐HPV‐positive women by GP5+/6+, 173 (45.9%) were *care*HPV‐positive, including 54.7% HPV16‐positive and 30.2% HPV18‐positive women.

**Conclusions:**

The final REACH‐Bhutan results show that screening for cervical cancer with self‐collection of samples and HR‐HPV testing, in addition to our previous report of achieving high participation, can also perform well to detect women with hHSIL+.

## Graphical abstract

The final results of REACH‐Bhutan study show that community‐based screening for cervical cancer with self‐collection of samples and HR‐HPV DNA testing can perform well as the initial step to detect and treat women with histologically proven high‐grade cervical lesions HSIL+, even in remote low‐resource settings.

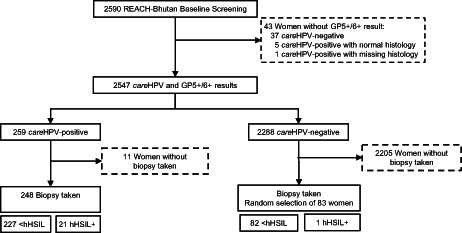

## 1 INTRODUCTION

Cervical cancer is the most frequent cancer among women in Bhutan,^1^ a country that is actively committed to cervical cancer prevention. In 2000, the Ministry of Health (MoH) of Bhutan launched a national cytology‐based screening program,^2^ followed by a national program of vaccination against human papillomavirus (HPV), reaching >90% coverage in girls aged 12–18 years in 2010.^3^ Cytology‐based screening coverage has been less widespread and unevenly implemented across the country,^4^ being particularly low in rural areas^5^ where the majority of the Bhutanese live. Follow‐up and treatment of screen‐positive women also presents a challenge.

High‐risk HPV (HR‐HPV)‐based screening has the advantage of extended screening intervals, self‐sampling, and test automation,^6^, ^7^, ^8^ providing an excellent opportunity to improve the coverage and cost‐effectiveness of cervical screening. Thus, in 2016, the Bhutan MoH and the International Agency for Research on Cancer (IARC) initiated the REACH‐Bhutan study, with the aim of assessing the feasibility, clinical performance, and challenges of screening for cervical cancer using the *care*HPV (Qiagen, Gaithersburg, MD) test on self‐collected vaginal samples in women 30–60 years of age from rural Bhutan. The design and implementation of the REACH‐Bhutan study, as well as the determinants of screening participation (most notably age and time taken to travel to the health center) and HPV positivity (most notably sexual behavior), have been reported previously, and showed that HPV‐based cervical cancer screening using self‐collection can achieve high coverage in rural Bhutan.^9^ Here, we discuss the clinical performance of the REACH‐Bhutan screening protocol. The cross‐sectional performance of *care*HPV on self‐collected samples was assessed against the gold standard of colposcopy and histologically proven high‐grade squamous epithelial lesions or worse (hHSIL+). Ascertainment bias was addressed by random biopsies among a subset of *care*HPV‐negative women and validation with a clinically validated HR‐HPV test that has shown good performance on self‐collected samples.^10^, ^11^

## 2 MATERIALS AND METHODS

### 2.1 Study population and recruitment

The design of the REACH‐Bhutan study has been previously described in detail.^9^ In brief, in April/May 2016, women aged 30–60 years were invited to attend cervical screening in 15 Basic Health Units (BHUs) providing primary health care in rural areas of Bhutan. Locally‐based health workers (HWs), accustomed to community mobilization, attended public information sessions in the villages covered by the preselected BHUs, to explain the benefits of screening for cervical cancer. Women who were known to be pregnant or who had undergone hysterectomy were not eligible. At the screening visit, women provided informed consent and completed a short electronic questionnaire. The study was approved by both the Research Ethical Board of the Bhutan Ministry of Health (REBH/PO/15/023) and the IARC Ethics Committee.

### 2.2 Sample collection, transportation, and laboratory analysis in Bhutan

Each participant provided a self‐collected cervicovaginal sample into *care*HPV universal collection medium (UCM) medium using a *care*Brush, as detailed previously.^9^ Specimen vials were transported to Jigme Dorji Wangchuck National Referral Hospital (JDWNRH) in Thimphu where an aliquot was tested on the *care*HPV platform (Qiagen Corporation, Gaithersburg, MD, USA) according to the manufacturer's instructions. The *care*HPV test is a signal amplification, rapid batch diagnostic test designed for the detection of the DNA of 13 HR‐HPV types (16, 18, 31, 33, 35, 39, 45, 51, 52, 56, 58, 59, and 68) and HPV66.^12^ *care*HPV results were given to each BHU to arrange colposcopy for all *care*HPV‐positive women, as well as for a random subset of *care*HPV‐negative women (~5 per BHU; total *n* = 83).

### 2.3 Cervical disease assessment

Mobile teams with a portable colposcope and cryotherapy equipment visited BHUs (or, exceptionally, closest hospitals), at which time all *care*HPV‐positive women (and the randomly selected subset of *care*HPV‐negative women) were invited to attend a follow‐up visit. Colposcopy was used to take biopsies from all suspicious areas in women with abnormal colposcopic findings or, in the absence of a specific suspicious area, randomly from 12 o'clock of the squamocolumnar junction. Treatment of colposcopy‐detected lesions was performed according to local protocols, primarily using cryotherapy on‐site (*n* = 119) or referral to closest hospitals for loop electrosurgical excision procedure (*n* = 214).

Histological reading of biopsies was first performed at the Department of Pathology at JDWNRH (T.T.), with a second expert reading (T.M.D.), both blinded to individual level HPV status (even if pathologists knew that most biopsies were obtained from HR‐HPV‐positive women). Results were reported according to LAST criteria ^13^ which was adopted as the reference diagnosis.

### 2.4 High‐risk HPV DNA detection and genotyping by GP5+/6+ PCR

Vials containing cellular material in *care*HPV UCM medium were shipped to the Department of Pathology at Amsterdam UMC location Vrije Universiteit Amsterdam, the Netherlands, where DNA was extracted using magnetic beads on a robotic system. The presence of human DNA in all specimens was confirmed by β‐globin PCR analysis as quality control for the extraction procedure and subsequent PCR. HR‐HPV positivity was assessed by GP5+/6+−mediated PCR^14^ followed by hybridization of PCR products in an enzyme immunoassay (EIA) with an oligoprobe cocktail for detection of 13 HR‐HPV types (16, 18, 31, 33, 35, 39, 45, 51, 52, 56, 58, 59, and 68). Genotyping of EIA‐positive samples was subsequently conducted by luminex hybridization of GP5+/6+−PCR products as described previously.^15^ EIA‐positive samples that failed to reveal a positive signal in the genotyping assay were designated as HR‐HPV type X (HPVX).

### 2.5 Statistical analyses

Standard screening indices of accuracy, including sensitivity, specificity, positive predictive value, negative predictive value, and their 95% confidence intervals (CI) were calculated for hHSIL+. Corrected indices were calculated after imputation of missing data for women who did not attend colposcopy.^16^, ^17^ In the corrected model, pseudo‐observations were created for women without a valid histology result and weighted by the probability of hHSIL+ among women with the same combination of *care*HPV and GP5+/6+ results who underwent colposcopy. This approach is valid under the assumption that lack of colposcopy was independent of the underlying hHSIL+ status, given the same combination of *care*HPV and GP5+/6+ test results (“missing at random”). All analyses were performed using STATA version 14.

## 3 RESULTS

**FIGURE 1:**
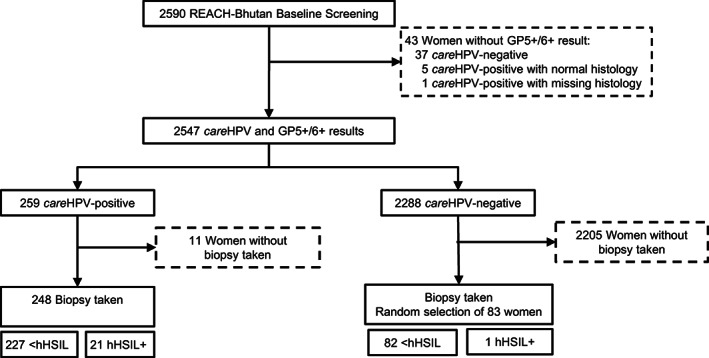
Flow chart of the study population, procedures, and outcomes. REACH‐Bhutan 2016–17. HPV, human papillomavirus; hHSIL+, histological high‐grade squamous intraepithelial lesions or worse.

Of 2590 women screened with *care*HPV in the REACH‐Bhutan study, 2547 with a valid HR‐HPV GP5+/6+ PCR‐EIA test result were included in the current analysis (Figure 1). Of the 43 excluded for lack of GP5+/6+ test, six were *care*HPV‐positive: five had histologically benign biopsy results and one did not undergo colposcopy (Figure 1). Adequate histology results were obtained from 331 of 332 women who underwent colposcopy, among whom 22 cases were diagnosed with hHSIL+, including one invasive squamous cell carcinoma. In total, 29 hHSIL+ cases (including seven pseudo‐observations among women without colposcopy) were included as outcomes in analyses (Table 1). Only corrected indices are shown, but crude indices, assuming that all women without valid histology had no hHSIL+ (albeit less methodologically valid), can also be calculated using the data presented in Table 1 (e.g., 21 out of 22 confirmed hHSIL+ were *care*HPV‐positive; crude sensitivity for *care*HPV = 95.5%, 95% CI 77.2%–99.9%) (see also Tables S1 and 2).

**TABLE 1:** Confirmed and imputed hHSIL+ among 2547 women aged 30–60 years, by combining *care*HPV and GP5+/6+ test results. REACH‐Bhutan 2016–17.

HR‐HPV test result		Women with biopsy		Women without biopsy		All women^a^	
*care*HPV	GP5+/6+	*N*	Confirmed hHSIL+	*N*	Imputed hHSIL+	*N*	hHSIL+
−	−	53	0	2031	0	2084	0
−	+	30	1	174	6	204	7
+	−	83	3	3	0	86	3
+	+	165	18^b^	8	1	173	19
		**331**	**22**	**2216**	**7**	**2547**	**29**

Abbreviations: HR‐HPV, high‐risk human papillomavirus; hHSIL+, histologically proven high‐grade squamous intraepithelial lesions or worse.

^a^ After correction for women without biopsy.

^b^ Including one invasive cervical cancer.

Table 2 shows screening indices of cross‐sectional clinical accuracy according to the two HR‐HPV DNA tests, namely *care*HPV performed locally and GP5+/6 PCR‐EIA performed in a reference laboratory. Screening test positivity was 10.2% and 14.8% for *care*HPV and GP5+/6 PCR‐EIA, respectively. HR‐HPV testing by GP5+/6+ PCR‐EIA showed higher sensitivity for hHSIL+ (89.7%) than *care*HPV (75.9%), but 95% CIs overlapped. Relative sensitivity of GP5+/6+ versus *care*HPV+ for hHSIL+ was 1.18 (95% CI = 0.93–1.50) *p* = 0.172. Negative predictive value was also slightly higher for GP5+/6+ PCR‐EIA (99.9%) than *care*HPV (99.7%), albeit with overlapping 95% CIs. Specificity, however, was lower for GP5+/6+ PCR‐EIA (86.1%) than *care*HPV (90.6%), relative specificity = 0.95 (95% CI = 0.93–0.97) *p* < 0.001, as was positive predictive value (6.9% vs. 8.5%).

**TABLE 2:** Performance of *care*HPV and GP5+/6+ to detect hHSIL+ among 2547 women aged 30–60 years.^a^ REACH‐Bhutan 2016–17.

HR‐HPV test	Test positivity (%)	Sensitivity (95% CI)	Specificity (95% CI)	PPV (95% CI)	NPV (95% CI)
*care*HPV	10.2	75.9 (56.5–89.7)	90.6 (89.4–91.7)	8.5 (5.4–12.6)	99.7 (99.4–99.9)
GP5+/6+	14.8	89.7 (72.6–97.8)	86.1 (84.6–87.4)	6.9 (4.5–9.9)	99.9 (99.6–100)

*Note*: Relative sensitivity of GP5+/6+ versus *care*HPV+ for hHSIL+ = 1.18 (95% CI = 0.93–1.50) *p* = 0.172.

Relative specificity of GP5+/6+ versus *care*HPV for hHSIL+ = 0.95 (95% CI = 0.93–0.97) *p* < 0.001.

Abbreviations: CI, confidence interval; HR‐HPV, high‐risk human papillomavirus; hHSIL+, histologically proven high‐grade squamous intraepithelial lesions or worse; PPV, positive predictive value; NPV, negative predictive value.

^a^ After correction for women without biopsy.

Type‐specific HR‐HPV prevalence, according to GP5+/6+ PCR‐based genotyping, is described in Table 3. Of the 2547 women tested, the most commonly detected HR‐HPV type was HPV16 (6.7%), followed by HPV18 (3.8%). HPV16 was detected in two‐thirds (14 of 21) hHSIL+ and in the single invasive cervical cancer. Of note, for 45 women who were GP5+/6+ PCR‐EIA‐positive, no specific individual HR‐HPV type was detected (HPVX). The proportion of GP5+/6+ PCR‐EIA‐positive cases that were positive by *care*HPV are also shown according to GP5+/6+−genotype in Table 3. Of 377 women positive for HR‐HPV by GP5+/6+ PCR‐EIA, 173 (45.9%) were also positive by *care*HPV. Fifty‐five percent of HPV16‐positive samples were *care*HPV‐positive. This proportion ranged from 15.6% of HPVX samples up to 100% of the few samples positive for HPV35, 39, and 68. Of note, *care*HPV positivity among GP5+/6+ PCR‐EIA‐positive samples varied strongly by optical density which may be used as a semiquantitative surrogate for HR‐HPV viral load, being 20.6%, 36.5%, and 80.8% for tertiles of low, medium, and high optical densities, respectively.

**TABLE 3:** Prevalence of overall HPV types among 2547 women aged 30–60 years. REACH‐Bhutan, 2016–2017.

	GP5+/6+ positive				*care*HPV‐positive among GP5+/6+ positive	
	Single *n*	Multiple *n*	Total *n* (%)		*n*	% (95% CI)
HR‐HPV−	–	–	2170	85.2		
HR‐HPV+	293	84	377	14.8	173	45.9 (40.8–51.1)
HR‐HPV+, by optical density^a^						
Low			126		26	20.6 (13.9–28.8)
Medium			126		46	36.5 (28.1–45.6)
High			125		101	80.8 (72.8–87.3)
HR‐HPV+, by type						
16	106^b^ ^,^ ^c^	66^b^	172	6.7	94	54.7 (46.9–62.2)
18	60	36^b^	96	3.8	29	30.2 (21.3–40.4)
31	5	4	9	0.4	8	88.9 (51.8–99.7)
33	13^b^	5	18	0.7	10	55.6 (30.8–78.5)
35	1	1	2	0.1	2	100 (15.8–100)
39	2	2	4	0.2	4	100 (39.8–100)
45	6	4	10	0.4	4	40.0 (12.2–73.8)
51	9	6	15	0.6	14	93.3 (68.1–99.8)
52	5	10	15	0.6	14	93.3 (68.1–99.8)
56	10	9	19	0.7	13	68.4 (43.4–87.4)
58	6^b^	7^b^	13	0.5	12	92.3 (64.0–99.8)
59	16	20	36	1.4	19	52.8 (35.5–69.6)
68	1	1	2	0.1	2	100 (15.8–100)
X	45^b^	0	45	1.8	7	15.6 (6.5–29.5)

Abbreviations: CI, confidence interval; HR‐HPV, high‐risk human papillomavirus.

^a^ Tertiles: optical density: low = 0.30–0.75; medium = 0.76–2.21; and high = 2.22–4.01.

^b^ Including GP5+/6+−positive hHSIL: HPV16 (*n* = 11), HPV16/18 (1), HPV16/58 (2), HPV33 (1), HPV58 (2), and HPVX (1)

^c^ Including one HPV16‐positive cervical cancer.

## 4 DISCUSSION

In addition to achieving high acceptability in rural Bhutan as we previously reported, these final REACH‐Bhutan results show that HPV‐based screening for cervical cancer with self‐collection of vaginal samples can achieve high performance in detecting women with hHSIL+. Our report adds to several existing assessments of HPV testing in self‐collected samples in underserved populations in low‐ or middle‐resource settings,^6^, ^18^, ^19^, ^20^, ^21^, ^22^ including those based upon *care*HPV testing.^23^, ^24^

Although *care*HPV was associated with a slightly lower sensitivity than the reference PCR‐based HPV test, GP5+/6+ PCR‐EIA, the performance of self‐collected *care*HPV to detect hHSIL+ in our study, with its 76% sensitivity and 91% specificity, is consistent with that estimated by a 2017 meta‐analysis of four *care*HPV evaluations (74% sensitivity and 88% specificity),^23^ and with a larger recently published report of self‐collected *care*HPV screening in China (73% sensitivity and 97% specificity).^24^

Previous studies have shown that, similar to other signal amplification methods, for instance Hybrid Capture 2 (HC2, Qiagen),^11^ *care*HPV has slightly lower sensitivity and higher specificity against cervical intraepithelial neoplasia grade 2 or worse (CIN2+) when performed on self‐collected versus clinician‐collected samples in the same study,^12^, ^23^, ^25^, ^26^ and that differences in sensitivity in between *care*HPV and HC2 may be bigger for self‐collected than clinician‐collected samples.^25^ Nevertheless, *care*HPV on self‐collected samples has been shown to remain more sensitive than either Pap tests^26^ or visual acetic acid (VIA) inspection^12^, ^25^, ^26^, ^27^ in the settings in which it has been evaluated head‐to‐head. Furthermore, the wider advantages of self‐collected over clinician‐collected sampling in terms of feasibility and acceptability to improve coverage^9^, ^11^ are expected to be more important for impact on cervical cancer prevention at a population level than small losses in test sensitivity.

There is a wider literature describing the performance of *care*HPV testing on clinician‐collected cervical specimens, which has also shown greater cross‐sectional sensitivity for CIN2+ than either VIA,^12^, ^26^, ^28^, ^29^, ^30^ or Pap tests,^26^, ^31^ and resulted in significantly higher CIN2+ yields than VIA or liquid‐based cytology in a large randomized control trial (>15,000 women tested for *care*HPV in rural China^32^). On the contrary, clinician‐collected *care*HPV has been associated with slightly lower sensitivity to detect CIN2+ versus HC2^27^, ^28^, ^31^ or PCR assays, for example Sansure (Sansure Biotech Inc.),^25^ INNO‐LiPA (Innogenetics N. V.),^35^ or GP5+/6+ PCR‐EIA.^32^

In the REACH‐Bhutan study on self‐collected samples, *care*HPV was also associated with lower sensitivity than GP5+/6+ PCR‐EIA. Indeed, we retested all REACH‐Bhutan samples with a reference PCR test for a number of reasons. Firstly, to improve the imputation of CIN2+ among *care*HPV‐negative women (see below). In addition, by performing full HR‐HPV genotyping on GP5+/6+ PCR‐EIA‐positive samples, we were able to investigate *care*HPV sensitivity according to HR‐HPV genotype. Overall, we found 46% of GP5+/6+ PCR‐based HR‐HPV‐positives to be *care*HPV‐positive from the same sample. This compares to 55% of HR‐HPV positives from the only previous comparison of *care*HPV with a PCR assay according to genotype (albeit using the more analytically sensitive INNO‐LiPA assay).^33^ With respect to the most carcinogenic HPV types relevant for cervical cancer screening, we found that 55% of HPV16‐positives and 30% of HPV18‐positives by GP5+/6+ PCR‐based genotyping were also detected by *care*HPV, which compares with 46% of HPV16 and 49% of HPV18 INNO‐LiPA–positive samples, respectively.^33^ Of 45 samples that were positive for the HR‐HPV GP5+/6+ PCR‐EIA cocktail probe, but for which no specific HR‐HPV genotype could be detected, only two (4%) were *care*HPV‐positive, suggesting that these samples contain infections not easily identified by either assay. The one HPVX with hHSIL was investigated and found to be HPV66‐positive which is not considered as a HR‐HPV type. Furthermore, *care*HPV positivity was strongly related to the strength of the semiquantitative GP5+/6+ PCR‐EIA optical density signal, a surrogate for HR‐HPV viral load. Of note, the distribution of HR‐HPV types in the REACH‐Bhutan sample of unvaccinated rural women aged 30–60 years, characterized by a strong predominance of HPV16, followed by HPV18 and HPV59, is comparable with that reported in an urban sample of unvaccinated women in the Bhutanese capital, Thimphu.^34^

hHSIL is considered the gold standard diagnosis for cervical precancerous lesions. However, verification bias may occur in histology when biopsies are not taken from all screened women. Here, we tried to overcome this issue by taking biopsies from a proportion of *care*HPV‐negative women, by submitting all biopsies to specialist review according to recommended LAST criteria,^13^ and by imputing underlying hHSIL+ in the few women with no histological reading, informed also by GP5+/6+ HPV testing results. Nevertheless, the number of *care*HPV‐negative women that were randomly recalled for colposcopy (*n* = 83), and the number of observed hHSIL+ with discordant *care*HPV and GP5+/6+ PCR‐EIA results was small, and so the accuracy of imputation of hHSIL+ prevalence to *care*HPV‐negative women without colposcopy is a limitation of our study.

A number of successes in the implementation of this screening initiative should be highlighted. Firstly, we previously reported on the high acceptability and feasibility of self‐collected samples in the rural Bhutanese population.^9^ With this report, we can also now add high completion rates for colposcopy and treatment among HR‐HPV‐positive women in Bhutan, showing that this can also be achieved among women in remote areas. Only 11 of 264 (4%) *care*HPV‐positive women did not attend colposcopy and undergo a biopsy. This low rate of loss to follow‐up is expected to be related to the high trust of Bhutanese population in their public health system, and the close interaction with local health workers. Of course, the loss to follow‐up in this well‐supported and punctual research study may be less than in a more widespread government campaign in Bhutan, if the follow‐up of screen‐positive women is not carefully planned and supported. Similarly, our present findings cannot necessarily be expected to be representative of other settings outside Bhutan. Indeed, this number of 4% compares with 25%–30% loss to follow‐up in some other experiences of recalling HPV‐positive women in low/medium‐income countries.^30^, ^35^, ^36^

On the contrary, we also encountered certain technical challenges when implementing *care*HPV testing, as might be expected, it being the first experience in Bhutan. For instance, as previously reported,^9^ there continued to be considerable wastage through invalid *care*HPV runs, even after validation of the initial training course. Indeed, lab implementation problems for *care*HPV were first noted for REACH‐Bhutan,^9^ but similar issues have since been mentioned in other reports,^30^, ^37^, ^38^, ^39^ even leading to the active development of a statistical model for quality assurance of *care*HPV contamination issues.^40^ Nevertheless, many other, indeed larger, experiences of successful implementation of *care*HPV have been reported without any mention of laboratory issues, including in Africa,^29^, ^41^, ^42^ in Latin America,^43^ in India,^44^ but most notably in China.^24^, ^28^, ^32^, ^45^, ^46^, ^47^, ^48^

Overall, the findings of the REACH‐Bhutan study highlight the potential for cervical cancer screening programs in low‐resource settings based on self‐collection of samples for HR‐HPV DNA testing. This is an approach that has been strongly endorsed by latest WHO guidelines for screening and treatment of cervical precancer lesions for cervical cancer prevention.^7^ This recommendation was informed by an IARC evaluation of cervical cancer screening methods,^8^ showing that the use of vaginal samples collected by women themselves can achieve a similar sensitivity and specificity for the detection of CIN2+ or CIN3+, at least when PCR‐based assays are used. Indeed, in addition to being implemented as the primary program in largely unscreened populations in low‐resource settings, self‐collection is increasingly being evaluated as a primary modality in organized HPV‐based programs in higher income settings.^10^, ^49^, ^50^ Self‐sampling may thus have a future role in the recently initiated policy of the Bhutanese government (https://www.moh.gov.bt/hspd/health‐flagship) to shift their entire national cytology‐based cervical cancer screening program to primary testing for HPV DNA.

## AUTHOR CONTRIBUTIONS

**Gary Clifford:** Conceptualization (equal); formal analysis (equal); funding acquisition (equal); methodology (equal); project administration (equal); supervision (equal); writing – original draft (equal); writing – review and editing (equal). **Iacopo Baussano:** Conceptualization (equal); funding acquisition (equal); methodology (equal); supervision (equal); writing – review and editing (equal). **DaniÃ«lle A.M. Heideman:** Data curation (equal); formal analysis (equal); validation (equal); writing – review and editing (equal). **Sangay Tshering:** Data curation (equal); writing – review and editing (equal). **Tashi Choden:** Data curation (equal); writing – review and editing (equal). **Fulvio Lazzarato:** Data curation (equal); software (equal); writing – review and editing (equal). **Vanessa Tenet:** Data curation (equal); formal analysis (equal); software (equal); visualization (equal); writing – review and editing (equal). **Silvia Franceschi:** Conceptualization (equal); funding acquisition (equal); methodology (equal); supervision (equal); writing – review and editing (equal). **Teresa Darragh:** Data curation (equal); validation (equal); writing – review and editing (equal). **Tashi Tobgay:** Data curation (equal); writing – review and editing (equal). **Ugyen Tshomo:** Conceptualization (equal); funding acquisition (equal); methodology (equal); supervision (equal); writing – review and editing (equal).

## FUNDING INFORMATION

This work was supported by the Bill and Melinda Gates Foundation (grant number OPP1053353).

## CONFLICT OF INTEREST STATEMENT

D.A.M.H. is minority shareholder of Self‐screen B.V., a spin‐off company of VUmc; Self‐screen B.V. develops, manufactures and licenses high‐risk HPV and methylation marker assays for cervical cancer screening and holds patents on these tests. The other authors declare no conflicts of interest.

## ETHICS STATEMENT

The present study had the approval of both the Research Ethical Board of the Bhutan Ministry of Health and the IARC Ethics Committee.

## DISCLAIMER

Where authors are identified as personnel of the International Agency for Research on Cancer/World Health Organization, the authors alone are responsible for the views expressed in this article and they do not necessarily represent the decisions, policy, or views of the International Agency for Research on Cancer/World Health Organization.

## Supporting information


Table S1.
Table S2.

## Data Availability

No additional data are available.
