# Supplementary material for: Human papillomavirus testing on self‐collected samples to detect high‐grade cervical lesions in rural Bhutan: The REACH‐Bhutan study
Source: Cancer Med. 2023 Mar 31;12(10):11828–37. doi: 10.1002/cam4.5851 (PMC10619475; doi:10.1002/cam4.5851)
Supplement: Supplementary file 1 — Table S1. Table S2. [file CAM4-12-11828-s001.docx]

**SUPPLEMENTARY TABLE 1** Confirmed hHSIL+ among 2547 women aged 30–60 years by *care*HPVtest results (without imputation for women without biopsy). REACH-Bhutan, 2016–17

| HR-HPV test result |  | Women with biopsy | |  | Women without biopsy | |  | All women | |
| --- | --- | --- | --- | --- | --- | --- | --- | --- | --- |
| *care*HPV |  | N | Confirmed hHSIL+ |  | N | Confirmed hHSIL+ |  | N | hHSIL+ |
| - |  | 48 | 1 |  | 2240 | 0 |  | 2288 | 1 |
| + |  | 248 | 21^†^ |  | 11 | 0 |  | 259 | 21 |
|  |  | 296 | 22 |  | 2251 | 0 |  | 2547 | 22 |

Abbreviations: HPV, human papillomavirus; hHSIL+, histologically proven high grade squamous intraepithelial lesions or worse.

^†^Including one invasive cervical cancer.

**SUPPLEMENTARY TABLE 2** Crude performance of *care*HPV to detect hHSIL+ among 2547 women aged 30–60 years (without imputation for women without biopsy). REACH-Bhutan 2016–17

| HR-HPV test | Test positivity (%) | Sensitivity  (95% CI) | Specificity  (95% CI) | PPV  (95% CI) | NPV  (95% CI) |
| --- | --- | --- | --- | --- | --- |
| *care*HPV | 10.2 | 95.5 (77.2–99.9) | 90.6 (89.4–91.7) | 8.1 (5.1–12.1) | 100 (99.8–100) |

Abbreviations: CI, confidence interval; HR-HPV, high-risk human papillomavirus; hHSIL+, histologically proven high-grade squamous intraepithelial lesions or worse; PPV, positive predictive value; NPV, negative predictive value.
